# Zilver PTX RCT mortality analysis: no difference in long-term mortality rate for Zilver PTX drug-eluting stent compared to PTA/BMS

**DOI:** 10.1186/s42155-019-0069-x

**Published:** 2019-07-27

**Authors:** Michael D. Dake, Gary M. Ansel, Aaron E. Lottes

**Affiliations:** 1 The University of Arizona, Tucson, AZ USA; 2 Department of Medicine, Ohio Health / Riverside Methodist Hospital, Columbus, OH USA; 3 Cook Research Incorporated, West Lafayette, IN USA

To the Editor,

In a recent meta-analysis by Katsanos et al., (Katsanos et al., 2018) the authors report an increased risk of death following treatment with a paclitaxel-coated balloon or a paclitaxel-eluting stent in the femoropopliteal artery. The authors included the published intent-to-treat data from the Zilver PTX randomized controlled trial (RCT) (Dake et al., 2011; Dake et al., 2013; Dake et al., 2016) in their analysis; however, these data do not account for all patients who received the Zilver PTX Drug-Eluting Stent (DES). When evaluating mortality potentially related to paclitaxel, it is important to analyze all patients who were treated with a DES. Katsanos et al. (Katsanos et al., 2018) did not have access to the patient-level data for the Zilver PTX RCT, limiting the validity of the analysis.

In the Zilver PTX RCT, after obtaining IRB approval and written informed consent, patients with symptomatic peripheral artery disease were initially randomized to percutaneous transluminal angioplasty (PTA) or stent placement with the DES. In cases where PTA failed acutely, patients underwent a secondary randomization to treatment with either the DES or a bare metal stent (BMS). Results through 5 years from the primary and secondary randomizations have been published (Dake et al., 2011; Dake et al., 2013; Dake et al., 2016) and demonstrate sustained safety and clinical durability in comparison with standard endovascular treatments. (Dake et al., 2016)

The study protocol also allowed optimal PTA patients who required reintervention within the first year post-procedure to cross over to treatment with the DES (i.e., long-term PTA failure, *n* = 30). In addition, one patient who had acute PTA failure and was treated with a BMS also required reintervention within the first year post-procedure and was treated with the DES. Therefore, an additional 31 patients who failed their initial treatment within the first year received a DES, which resulted in an as-treated patient population with a total of 336 patients treated with the DES and 143 patients treated with PTA and/or BMS. In previous publications, these 31 cross-over patients were analyzed per their initial assigned treatment groups (i.e., PTA or BMS) for evaluation of the study endpoints. Therefore, these 31 cross-over patients have not been previously analyzed as DES patients. Fig. 1 presents the treatment of patients in the RCT, which represents the actual treatment received in comparison to the intent-to-treat patient accountability previously published by Dake et al. (Dake et al., 2016) The patient-level data used for the analyses presented here is available on the following website: https://www.cookmedical.com/peripheral-intervention/paclitaxel/.

**Fig. 1:**
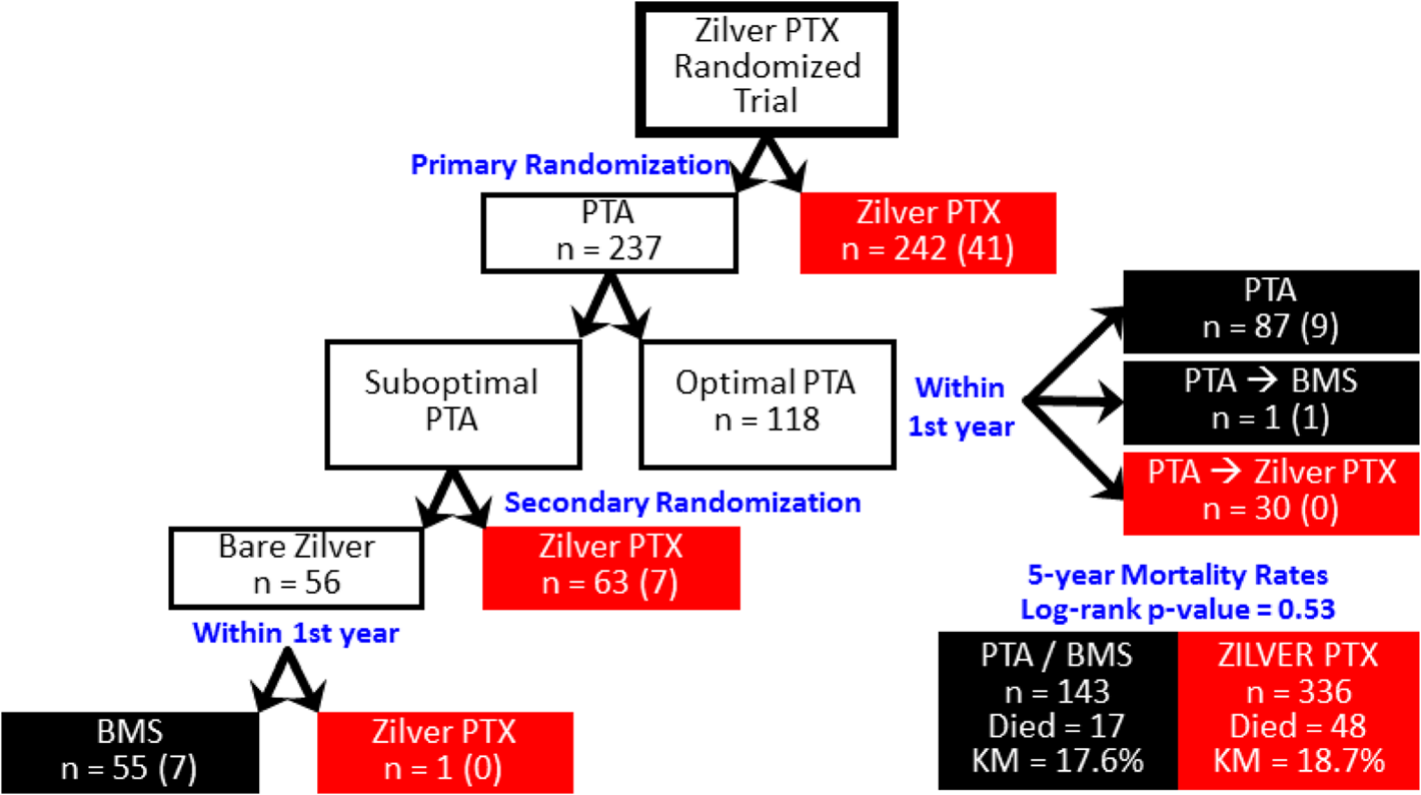
Flow chart for the Zilver PTX RCT showing long-term failures and 5-year mortality outcomes

The current analysis evaluates mortality in all patients treated with the DES regardless of the patients’ original treatment assignments. All-cause mortality for the group of patients who were treated with the DES was compared to the group of patients who were treated with PTA and/or BMS. There were 48 deaths in the DES group and 17 deaths in the PTA/BMS group. All deaths were adjudicated by an independent clinical events committee, and none were determined to be procedure- or device-related. Causes of death included cardiovascular (*n* = 24), cancer (*n* = 18), pulmonary (*n* = 8), stroke (*n* = 3), trauma/accident (n = 2), gastrointestinal (*n* = 1), and multiple/unknown (*n* = 9). There were no significant differences between the DES and PTA/BMS groups in causes of death. Kaplan-Meier estimates for 5-year all-cause mortality were 18.7% for the DES group and 17.6% for the PTA/BMS group (*p* = 0.53). These results demonstrate no difference in the long-term mortality rate for the Zilver PTX DES compared to PTA/BMS.

The concerns raised by the meta-analysis are serious; FDA’s Letter to Health Care Providers (U.S. Food and Drug Administration, 2019) underscored the potential concern and highlighted the need for continued investigation to evaluate the report of an increased mortality rate with the use of paclitaxel-coated endovascular devices. Our ongoing analyses of global long-term Zilver PTX DES mortality data will be further described in a future publication. In collaboration with regulatory agencies, the clinical community, and device manufacturers, we look forward to a greater understanding of the data surrounding paclitaxel-coated devices so we can provide patients with optimal care.

## Data Availability

The dataset generated and analyzed during the current study are available on the following website: https://www.cookmedical.com/peripheral-intervention/paclitaxel/.

## Abbreviations

BMS: Bare metal stent
DES: Drug-eluting stent
PTA: Percutaneous transluminal angioplasty
RCT: Randomized controlled trial
